# Laboratory variables‐based artificial neural network models for predicting fatty liver disease: A retrospective study

**DOI:** 10.1515/med-2024-1031

**Published:** 2024-09-13

**Authors:** Panpan Lv, Zhen Cao, Zhengqi Zhu, Xiaoqin Xu, Zhen Zhao

**Affiliations:** Department of Clinical Laboratory, Minhang Hospital, Fudan University, Shanghai, China

**Keywords:** fatty liver disease, artificial neural network, model, prediction, laboratory variables

## Abstract

**Background:**

The efficacy of artificial neural network (ANN) models employing laboratory variables for predicting fatty liver disease (FLD) remains inadequately established. The study aimed to develop ANN models to precisely predict FLD.

**Methods:**

Of 12,058 participants undergoing the initial FLD screening, 7,990 eligible participants were included. A total of 6,309 participants were divided randomly into the training (4,415 participants, 70%) and validation (1,894 participants, 30%) sets for developing prediction models. The performance of ANNs was additionally tested in the testing set (1,681 participants). The area under the receiver operating characteristic curve (AUROC) was employed to assess the models’ performance.

**Results:**

The 18-variable, 11-variable, 3-variable, and 2-variable models each achieved robust FLD prediction performance, with AUROCs over 0.92, 0.91, and 0.89 in the training, validation, and testing, respectively. Although slightly inferior to the other three models in performance (AUROC ranges: 0.89–0.92 vs 0.91–0.95), the 2-variable model showed 80.3% accuracy and 89.7% positive predictive value in the testing. Incorporating age and gender increased the AUROCs of the resulting 20-variable, 13-variable, 5-variable, and 4-variable models each to over 0.93, 0.92, and 0.91 in the training, validation, and testing, respectively.

**Conclusions:**

Implementation of the ANN models could effectively predict FLD, with enhanced predictive performance via the inclusion of age and gender.

## 1 Introduction

Fatty liver disease (FLD) is an increasingly prevalent global health issue, affecting over 25% of adults worldwide and posing a significant economic burden on society, the prediction and diagnosis of which is necessary for management and prognosis. However, early detection of FLD is challenging because of its silent and nonspecific symptomatology, compounded by limited technological capabilities for detection [1,2].

The current standard clinical workup for individuals suspected of or diagnosed with liver disease involves obtaining a comprehensive medical record, performing a thorough physical check, conducting laboratory tests, and interpreting imaging results [3]. Although these data modalities offer an abundance of information, their interpretation can be complex even for experienced clinicians. Hepatology is particularly prone to diagnostic ambiguities. Therefore, there is a need for advanced diagnostic approaches and improved technology to enhance the screening, early diagnosis, and subsequent intervention of FLD. Addressing these challenges is paramount alleviating the global burden of FLD and enhancing patient outcomes.

There have been several attempts to predict FLD. However, given its multifactorial nature, accurately predicting the occurrence of FLD using a single laboratory test parameter is unlikely. Ultrasonography (US) has been proposed as an initial screening modality for identifying steatosis in a specific cohort [4]. However, US has well-described limitations, particularly in its ability to detect focal liver lesions. These limitations pertain to a significant reliance on operator expertise, equipment standards, and patient physique [5]. Currently, liver biopsy is considered the diagnostic gold standard for assessing fatty infiltration of the liver and stratifying patients. Nonetheless, this invasive and costly method has its drawbacks, including the potential for side effects, sampling errors, and a lack of agreement among different observers [5]. Consequently, there is a growing demand for non-invasive or minor-invasive predictive models of FLD.

Studies are trying to find new markers or combined diagnoses for the early diagnosis of FLD to improve the sensitivity and clinical application. The construction of a prediction model to effectively identify high-risk groups and carry out targeted interventions is helpful not only for disease treatment but also for avoiding unnecessary excessive examinations and improving the utilization rate of medical resources.

**Figure 1:**
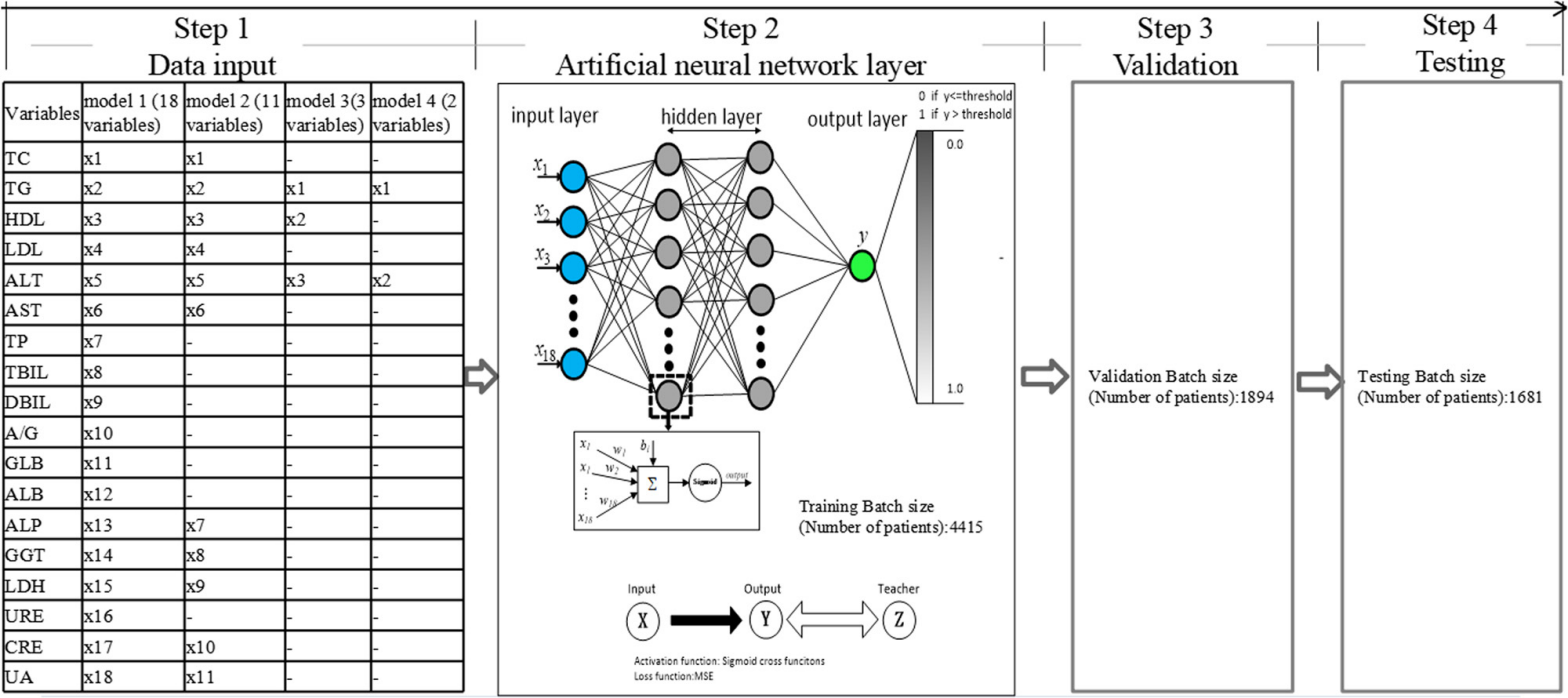
Data flowchart and architecture of the feed-forward ANN for the proposed ANN of this study. The ANN comprises an input layer, two hidden layers, and an output layer. Within each layer, a multitude of neurons, represented by solid circles, is present. The interconnections between these neurons are visually denoted by black lines.

Given the intricate nature of liver diseases and the often non-linear relationships between various variables and clinical outcomes, artificial neural network (ANN) has gained prominence in the past decade, particularly in medical model classification and assessment [3]. ANN is a robust machine learning model inspired by the neuroanatomy of the brain that is capable of non-linear statistical analysis. Comprising interconnected processing neurons with weighted connections, ANN forms a network structure that consists of an input layer, an output layer, and one or more hidden layers (Figure 1). Through training on extensive medical data, ANNs have the ability to extract hidden properties, offering a novel approach to effective discrimination [6]. In contrast, traditional statistical algorithms lack this adaptability, relying on explicit expressions of relationships [7]. In the case of diagnosing a specific condition, an ideal screening test should be simple to apply to the target population [8]. Therefore, prediction models of FLD based solely on laboratory test results plus demographic factors obtained at the time of examination using ANN were constructed in this study, so as to explore whether ANNs could serve as a promising strategy for FLD prediction from readily available tests. To validate this hypothesis, we evaluated and compared the predictive performance of eight different ANNs based on four distinct serum panels with and without the inclusion of demographic factors.

## 2 Materials and methods

### 2.1 Patients

Patients who had undergone initial screening for fatty liver examination at the physical examination center of Minhang Hospital, Fudan University (Shanghai, China) from 2021 to 2022 were enrolled in this study. Individuals with incomplete screening processes, known liver-related diseases, such as viral hepatitis (HCV, HBV), or suspected cases of fatty liver identified by US were excluded.

To construct and evaluate the performance of ANNs, the population between January 1, 2021, and July 31, 2022, included in this study was randomly divided into two subsets with 70% of patients (*N* = 4,415) in the training set and the remaining 30% (*N* = 1,894) in the validation set; 1,681 additional patients were enrolled between August 1, 2022, and December 31, 2022, to constitute the testing set, which was used to further assess the efficacy of the established models.

Blood tests were conducted on the day of the physical examination, following the detailed protocol outlined below. Two expert hepatologists used the previously published criteria [4,9,10] for the FLD diagnosis. In cases where there was a disagreement between the two hepatologists, a third experienced hepatologist was consulted to provide a judgment.

### 2.2 Clinical data acquisition

The clinical data used in our study were collected from both FLD and non-FLD patients, including 39 blood variables, which were determined at the time of screening and used in our proposed models. Thirty-nine blood variables (18 clinical chemistry variables and 21 complete blood counts) were as follows: (1) 18 clinical chemistry variables: total cholesterol (TC), triglyceride (TG), high-density lipoprotein cholesterol (HDL-c), low-density lipoprotein cholesterol (LDL-c), alanine aminotransferase (ALT), aspartate aminotransferase (AST), total protein (TP), total bilirubin (TBIL), direct bilirubin (DBIL), serum albumin (ALB), globulin (GLB), A/G, alkaline phosphatase (ALP), gamma-glutamyltransferase (GGT) level, lactic dehydrogenase (LDH), ureophil (URE), creatinine (CRE), and serum uric acid (UA), and (2) 21 complete blood counts: erythrocyte count, hemoglobin, neutrophil, lymphocytes, monocyte, acidophilic cell, basophilic granulocyte, neutrophil ratio%, ratio of lymphocytes%, monocyte%, acidophilic cell%, basophilic granulocyte%, monocyte%, peripheral platelet count, packed cell volume, mean corpuscular hemoglobin, mean corpuscular hemoglobin concentration, red cell distribution width SD, red cell distribution width CV, mean platelet volume, and platelet distribution width. All FLD and non-FLD patients were identified by the abdominal US. Clinical chemistry tests were performed using a Cobas c 702 Analyzer (Cobas, Germany), and the complete blood counts were measured on a BC-6000 Analyzer (Mindray, China). The prediction models with optimized variables were utilized to identify high-risk FLD patients, allowing for individualized health treatment in FLD patients.

### 2.3 Statistical analysis

Continuous variables were presented as the mean ± standard deviation. Comparisons between groups of quantitative variables were made by the one-way analysis of variance or Welch test, when appropriate. Statistical analysis was performed using the SPSS software version 20 (SPSS Inc., USA) and statistical significance was reported as *p*-values below 0.05.

### 2.4 Model development

The ANNs’ training and performance evaluation were conducted using Matlab with the neural pattern recognition application of the neural network toolbox from MathWorks, the Netherlands. Initially, we constructed models using only blood variables. A four-layer feed-forward neural network with a single output neuron was constructed (Figure 1). The backpropagation of errors learning rule was employed, allowing the network’s internal variables to be adjusted over repeated training cycles to minimize the overall error [11]. The activation function, representing the ANN’s outcomes, produced continuous outputs within the interval from 0 to 1 [12], where 0 indicated non-FLD and 1 represented FLD. Signal propagation occurred from the input layer through two hidden layers before reaching the output layer (Figure 1). These layers were fully connected, meaning that any neuron in the upper layer was connected to all neurons in the lower layer. The ANN training process involved randomly dividing the dataset into a training set (70% of total patients) for determining the network’s architecture and establishing the weights between nodes. A validation set (30% of total patients) was then utilized to evaluate the ANN’s ability to predict the desired output. Lastly, the neural network’s performance was assessed using an independent testing set. During training, the connection weights between neurons were adjusted iteratively to minimize the overall error. Training ceased when the sum of squared errors reached a minimum [12,13,14]. The number of neurons in the input layer was determined by the input data, with an *n*-dimensional vector included. Denoting the input layer as *X*, the output of the hidden layer was given by *f*(*W*_1_ *X* + *b*_1_), in which *W*_1_ represented the weights, *b*_1_ denoted the biases, and function *f* commonly employed activation functions (sigmoid function) [15]. Data propagation between the hidden layer and output layer followed softmax regression. The output of the output layer was softmax(*W*_2_ *X*_1_ + *b*_2_), where *X*_2_ (equal to *f*(*W*_1_ *X* + *b*_1_)) represented the hidden layer’s output. The formulation for the four-layer ANN described above can be summarized as follows [15]:

(1) \[f(x)=G({b}^{(2)}+\text{}{W}^{(2)}(s({b}^{(1)}+\text{}{W}^{(1)}x))).]\]

The function *G* denotes the softmax function as described previously. Therefore, all variables of the ANN symbolize the connection weights and the bias between the layers, encompassing *W*_1_, *b*_1_, *W*_2_, and *b*_2_ [15].

### 2.5 Model evaluation

The model’s prediction performance was evaluated using a confusion matrix, area under the receiver operating characteristic curve (AUROC), and classification accuracy. The receiver operating characteristic curve (ROC) methodology, which is closely related to neural networks in classification applications, was employed [16]. The AUROC, a commonly used accuracy index, was calculated to assess the diagnostic accuracy, with values close to 1 indicating higher accuracy [8]. We assessed the weight of each variable by calculating the AUROC, respectively, to evaluate the prediction performance of the models. To test whereas ANNs, based on readily available and inexpensive variables, may add performance to the prediction of FLD, in the training group, four types of ANNs were developed for predicting FLD, that is, 18-variable model (model 1, including TC, TG, HDL-c, LDL-c, ALT, AST, TP, TBIL, DBIL, ALB, GLB, A/G, ALP, GGT, LDH, URE, CRE, and UA), 11-variable model (model 2, including ALT, AST, TG, TC, r-GT, LDL-c, HDL-c, ALP, LDH, CRE, and UA), 3-variable model (model 3, including ALT, TG, and HDL), and 2-variable model (model 4, including ALT and TG). These four models were selected based on their specific characteristics. Firstly, all 39 blood variables were used for training and the best subset of relevant parameters was identified for subsequent model building. The automatic classification process registered approximately 75% sensitivity for FLD and non-FLD, which demonstrated that the classification performance was poor. Because of the overall small effect on the classification performance, the 21 routine blood variables were abandoned. After subtracting of routine blood indicators, 18 serum variables were trained (18-variable model). However, some variables are not readily available, especially for basic hospitals, and are expensive in routine diagnosis and treatment. To select the most promising predictive variables and achieve the highest predictive accuracy for FLD prediction, we retained variables with AUROC greater than 0.6 as input for the model. It helped to assess the efficacy of the variables incorporated in the training set. Thereafter, any invalid variables for classification were eliminated, resulting in an 11-variable model. Finally, to identify inexpensive and readily available variables with the least amount of detection requirements to aid clinicians in predicting FLD, ensuring patients receive appropriate and accurate treatment while maximizing the utilization of medical resources, we traversed three variables and two variables from the 18 variables to determine the optimal combination. We systematically evaluated each combination’s AUROC of any 3-variable combinations, which constitute 816 possible combinations (*C* (18,3) = 18 × 17 × 16/(3!) = 816), and any 2-variable combinations, totaling 153 possible combinations (*C* (18,2) = 18 × 17/(2!) = 153). Subsequently, we selected the combination with the highest AUROC as the optimal configuration for both the 3-variable and 2-variable models. It helped in evaluating the effectiveness. This meticulous process enabled us to derive the performance metrics for both models based on their respective optimal combinations of variables. Consequently, we developed both a 3-variable model and a 2-variable model. The final weights of the variables for the 2-variable and 3-variable models were determined based on the AUROC of all the training, validation, and testing sets. We also calculated the sensitivity (SEN), specificity (SPE), accuracy (ACC), false positive rate (FPR), and positive predictive value (PPV). Moreover, to further analyze the potential value of incorporating additional easily collectible and demographic factors, such as age and gender, into the constructed models, we incorporated these two factors for advanced analyses, resulting in a 20-variable model (model 5, including the variables of 18-variable model plus age and gender), a 13-variable model (model 6, including the variables of 11-variable model plus age and gender), a 5-variable model (model 7, including the variables of 3-variable model plus age and gender), and a 4-variable model (model 8, including the variables of 2-variable model plus age and gender).

The confusion matrix (Table 1) was used to determine the relationship between the actual values and predicted values [17]. The horizontal and vertical coordinates of the receiver operating characteristic curve are represented using 1 − specificity and sensitivity, respectively:

(2) \[\text{Sensitivity}(\text{SEN})\hspace{.25em}=\text{TP}/(\text{TP}+\text{FN}),]\]

(3) \[\text{Specificity}(\text{SPE})\hspace{.25em}=\text{TN}/(\text{TN}+\text{FP}),]\]

**Table 1:** Confusion matrix representation

Reality	Positive	Negative
Predicted true (+)	TP (true positive)	TN (true negative)
Predicted false (−)	FP (false positive)	FN (false negative)

Other evaluation indexes are calculated as follows:

(4) \[\text{Accuracy}(\text{ACC})\hspace{.25em}=(\text{TP}+\text{TN})/(\text{TP}+\text{FP}+\text{TN}+\text{FN}),]\]

(5) \[\text{Positive predictive value}(\text{PPV})\hspace{.25em}=\text{TP}/(\text{TP}+\text{TN}).]\]

In light of the aforementioned evaluation indexes of the model, we introduced the indicators in the optimal FLD prediction model, by comparing the results of different modeling methods to determine the optimal modeling method for the research data.


**Ethics approval:** The research has been complied with all the relevant national regulations, institutional policies and in accordance with the tenets of the Helsinki Declaration, and has been approved by the Ethics Committee of Minhang Hospital, Fudan University (047-01K).
**Informed consent:** The review board of the Ethics Committee deemed the study exempt from review and waived the requirement for informed consent due to the utilization of only de-identified data.

## 3 Results

### 3.1 Patient characteristics

**Figure 2:**
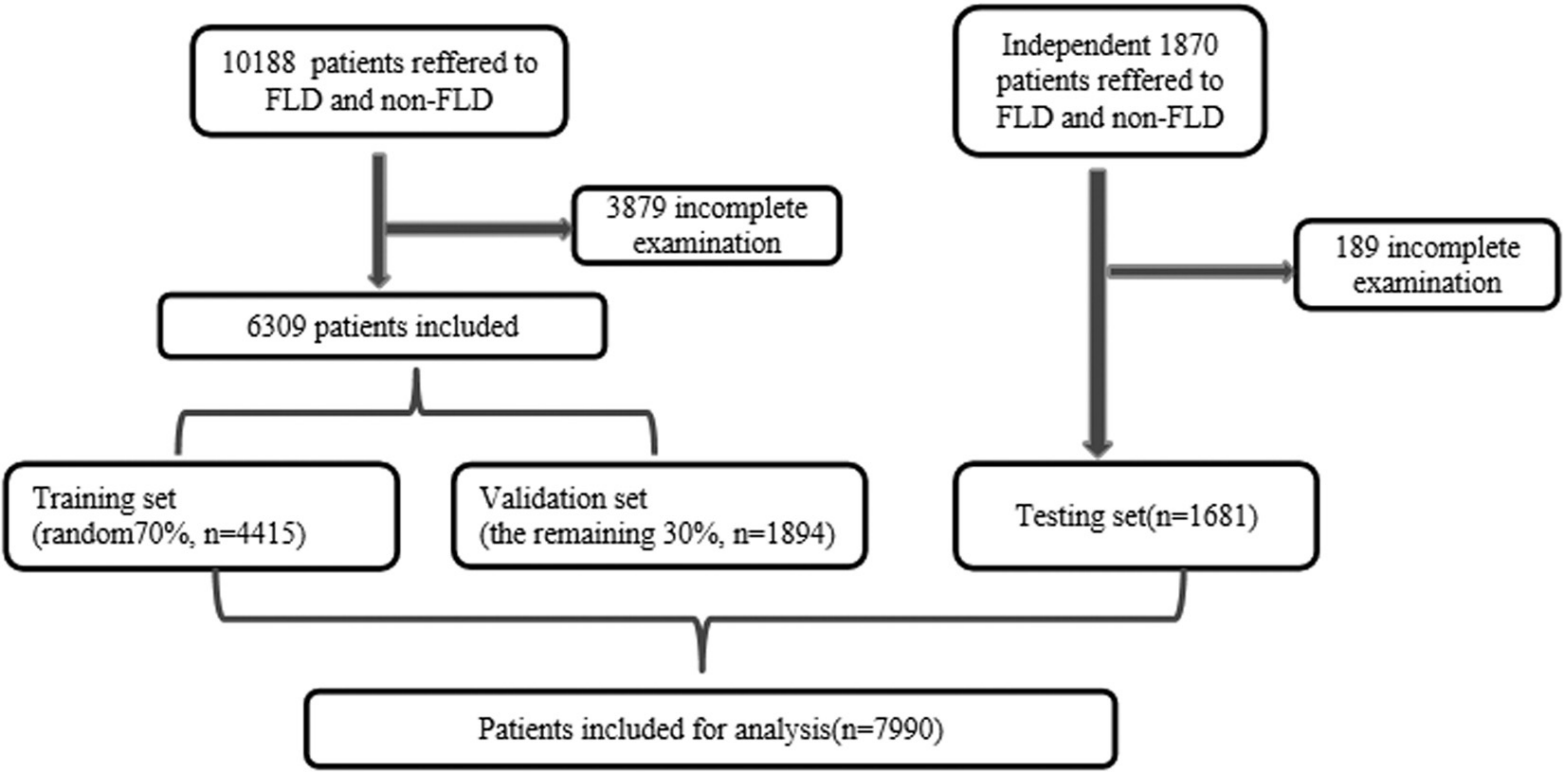
Workflow for patient screening.

A total of 12,058 participants who underwent initial fatty liver screening at Minghang Hospital, Fudan University between January 1, 2021, and December 31, 2022, were identified. Among them, 4,068 patients were excluded due to incomplete examination, resulting in a final sample size of 7,990 patients who fulfilled all inclusion criteria for model development (Figure 2). Of the overall subjects, 4,495 (56.3%) were patients with non-FLD, and the remaining 3,495 (43.7%) were FLD. Their mean age was 37.3 ± 8.5 years; 3,847 (48.1%) were male and 4,143 (51.9%) were female. A comparison between patients with and without FLD is presented in Table 2, revealing all variables (except for DBIL) to be statistically significant factors associated with FLD. Additional details regarding the distribution of variables of the FLD and non-FLD groups within the training, validation, and testing sets, are shown in Table 3. The training set comprised 4,415 subjects (1,717 FLD and 2,698 non-FLD), the validation set included 1,894 subjects (737 FLD and 1,157 non-FLD), and the testing set contained 1,681 subjects (1,041 FLD and 640 non-FLD). In the training set, all variables (except for DBIL) were found to be statistically significant factors associated with FLD. Variables (except for DBIL, A/G, and GLB) were found to be statistically significant factors associated with FLD in the validation set. Whereas, in the testing set, all variables were found to be statistically significant factors associated with FLD (Table 3).

**Table 2:** Comparing characteristics of patients with and without significant FLD

Variables	Reference range	FLD (*N* = 3,495)	Non-FLD (*N* = 4,495)	*p*-value
Male gender		2,615(74.8%)	1,231(27.4%)	<0.001
Median age (years)		39.9(±8.5)	35.3(±7.9)	<0.001
TC (mmol/L)	2.8–5.9	4.9(±0.9)	4.5(±0.8)	<0.001
TG (mmol/L)	0–2.3	2.4(±2.4)	1.1(±0.6)	<0.001
HDL (mmol/L)	0.9–1.68	1.1±(0.3)	1.6(±0.4)	<0.001
LDL (mmol/L)	<3.1	3.3(±0.8)	2.8(±0.7)	<0.001
ALT (U/L)	0–66	38.2(±29.5)	15.2(±13.7)	<0.001
AST (U/L)	0–40	24.7(±13.8)	17.5(±9.3)	<0.001
TP (g/L)	64–83	75.8(±3.8)	74.8(±3.9)	<0.001
TBIL (μmol/L)	2–20	11.9(±5.4)	11.1(±5.3)	<0.001
DBIL (μmol/L)	0–6	4.4(±1.6)	4.3(±1.6)	0.091
A/G	1.1–1.8	1.8(±0.3)	1.9(±0.3)	0.045
GLB (g/L)	29–33	27.4(±3.6)	26.6(±3.5)	<0.001
ALB (g/L)	35–50	48.4(±2.6)	48.2(±2.7)	0.001
ALP (U/L)	39–120	77.4(±19.9)	64.1(±22.1)	<0.001
GGT (U/L)	0–54	46.5(±41.5)	18.8(±19.4)	<0.001
LDH (U/L)	135–225	169.2(±28.5)	155.9(±23.9)	<0.001
URE (mmol/L)	1.7–8.3	4.8(±1.2)	4.6(±1.2)	<0.001
CRE (μmol/L)	20–110	78.3(±18.4)	67.6(±1.9)	<0.001
UA (μmol/L)	142–416	397.0(±90.1)	294.8(±77.6)	<0.001

Results are shown as the mean ± SD. *p*-values were calculated between the data of the FLD and the non-FLD. Statistical significance was reported as *p*-values below 0.05.

**Table 3:** Comparing characteristics of patients with and without significant FLD within a set (i.e., training, validation, or testing)

		Training set (*N* = 4,415)			Validation set (*N* = 1,894)			Testing set (*N* = 1,681)		
Variables	Reference range	FLD (*N* = 1,717)	Non-FLD (*N* = 2,698)	*p* ^1^-value	FLD (*N* = 737)	Non-FLD (*N* = 1,157)	*p* ^2^-value	FLD (*N* = 1,041)	Non-FLD (*N* = 640)	*p* ^3^-value
Male gender		1,280(74.5%)	798(29.6%)	<0.001	570(77.3%)	335(29.0%)	<0.001	765(73.5%)	98(15.3%)	<0.001
Median age (years)		39.0(±7.1)	35.4(±7.9)	<0.001	39.3(±7.3)	34.9(±7.9)	<0.001	41.7(±11.0)	35.7(±8.3)	<0.001
TC (mmol/L)	2.8–5.9	4.9(±0.9)	4.4(±0.8)	<0.001	4.8(±0.8)	4.4(±0.7)	<0.001	5.1(±0.9)	4.8(±0.9)	<0.001
TG (mmol/L)	0–2.3	2.5(±2.8)	1.1(±0.6)	<0.001	2.4(±1.8)	1.0(±0.6)	<0.001	2.3(±2.0)	1.0(±0.6)	<0.001
HDL (mmol/L)	0.9–1.68	1.1(±0.2)	1.5(±0.4)	<0.001	1.1(±0.3)	1.5(±0.3)	<0.001	1.2(±0.3)	1.6(±0.4)	<0.001
LDL (mmol/L)	<3.1	3.3(±0.8)	2.8(±0.7)	<0.001	3.2(±0.8)	2.8(±0.7)	<0.001	3.2(±0.8)	2.9(±0.8)	<0.001
ALT (U/L)	0–66	39.1(±30.6)	15.4(±15.2)	<0.001	37.5(±28.5)	14.6(±10.6)	<0.001	37.5(±28.5)	15.8(±11.7)	<0.001
AST (U/L)	0–40	25.6(±15.4)	17.8(±10.1)	<0.001	24.7(±12.8)	17.6(±8.8)	<0.001	23.4(±11.2)	16.3(±6.2)	<0.001
TP (g/L)	64–83	75.6(±3.8)	74.8(±3.9)	<0.001	75.5(±3.8)	75.0(±3.8)	0.005	76.2(±3.8)	74.4(±3.9)	<0.001
TBIL (μmol/L)	2-20	11.7(±5.3)	11.2(±5.4)	0.007	12.2(±5.9)	11.1(±5.2)	<0.001	12.0(±5.2)	10.6(±4.8)	<0.001
DBIL (μmol/L)	0–6	4.3(±1.5)	4.3(±1.6)	0.332	4.4(±1.6)	4.3(±1.6)	0.184	4.5(±1.6)	4.2(±1.5)	0.001
A/G	1.1–1.8	1.8(±0.3)	1.9(±0.3)	0.02	1.9(±0.3)	1.9(±0.3)	0.869	1.7(±0.3)	1.8(±0.3)	<0.001
GLB (g/L)	29–33	26.9(±3.5)	26.4(±3.5)	<0.001	26.8(±3.4)	26.6(±3.3)	0.217	28.6(±3.6)	27.4(±3.6)	<0.001
ALB (g/L)	35–50	48.7(±2.6)	48.3(±2.6)	<0.001	48.7(±2.6)	48.4(±2.7)	0.014	47.6(±2.5)	47.0(±2.5)	<0.001
ALP (U/L)	39–120	76.6(±19.2)	63.9(±21.9)	<0.001	77.6(±21.1)	64.7(±24.6)	<0.001	78.6(±20.2)	63.4(±17.2)	<0.001
GGT (U/L)	0–54	46.6(±44.3)	19.0(±20.9)	<0.001	46.9(±39.0)	18.0(±16.2)	<0.001	46.2(±38.3)	19.4(±17.7)	<0.001
LDH (U/L)	135–225	167.3(±27.6)	155.5(±23.6)	<0.001	168.1(±28.1)	156.0(±24.5)	<0.001	173.0(±30.0)	157.4(±23.5)	<0.001
URE (mmol/L)	1.7–8.3	4.8(±1.3)	4.6(±1.1)	<0.001	4.9(±1.1)	4.6(±1.2)	<0.001	4.7(±1.1)	4.5(±1.1)	<0.001
CRE (μmol/L)	20–110	78.2(±21.4)	68.1(±14.0)	<0.001	79.1(±15.2)	67.9(±14.1)	<0.001	77.8(±14.5)	65.1(±12.6)	<0.001
UA (μmol/L)	142–416	397.5(±90.8)	295.9(±76.7)	<0.001	397.8(±88.7)	296.3(±80.5)	<0.001	395.5(±89.9)	287.8(±75.8)	<0.001

Results are shown as the mean ± SD. The training set comprised 4,415 subjects (1,717 FLD and 2,698 non-FLD), the validation set included 1,894 subjects (737 FLD and 1,157 non-FLD), and the testing set contained 1,681 subjects (1,041 FLD and 640 non-FLD). *p* ^1^, *p* ^2^, and *p* ^3^ were calculated among the data of patients with and without significant FLD within the training, validation, and testing sets, respectively. Statistical significance was reported as *p*-values below 0.05.

## 4 Model performance

### 4.1 Training group

**Figure 3:**
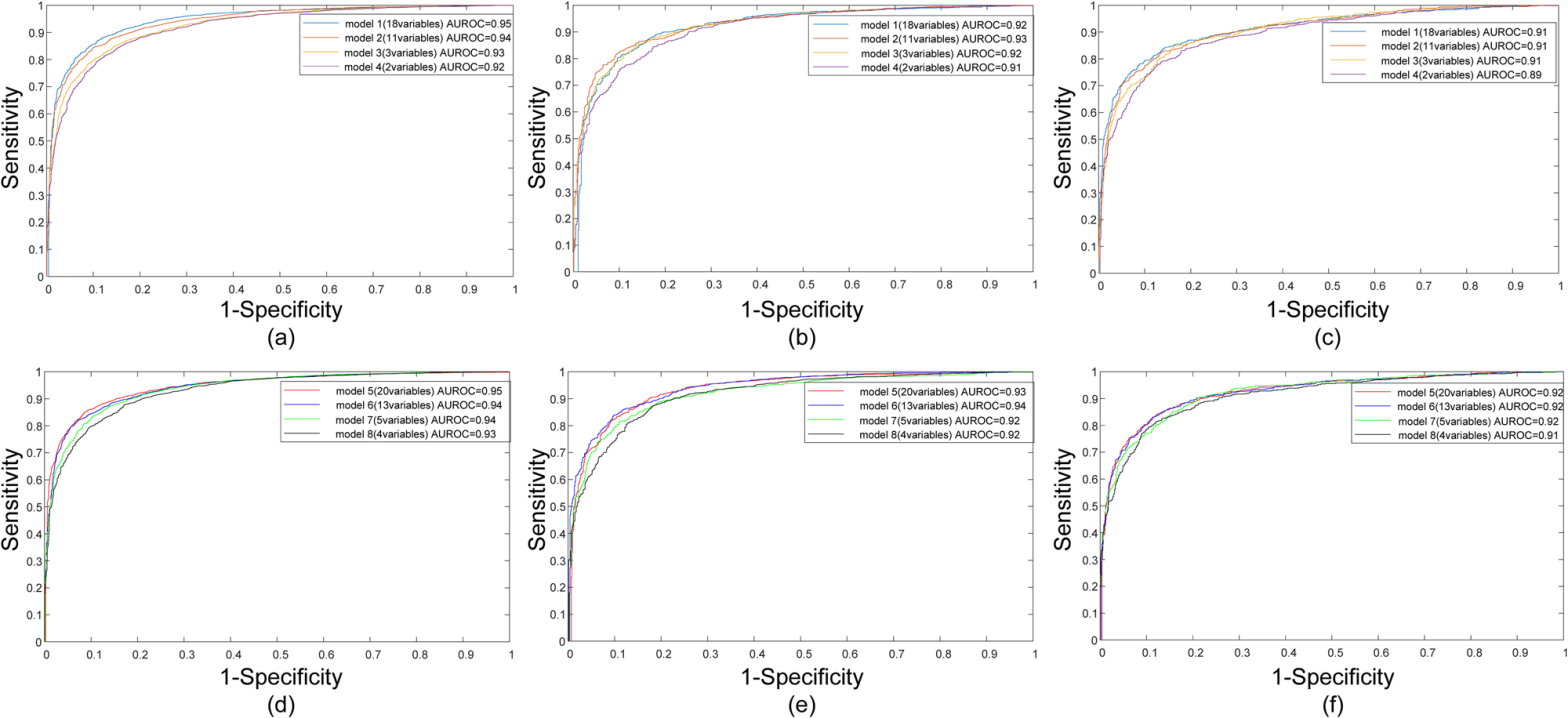
The ROC curves of the eight different models for the prediction of FLD in the training set (a and d), the validation set (b and e), and the testing set (c and f).

Through the training on the training set, the four models – 18-variable, 11-variable, 3-variable, and 2-variable, all achieved a very robust performance on FLD prediction, with AUROCs of 0.95, 0.94, 0.93, and 0.92, respectively. When age and gender were incorporated, the AUROC for the four models – 20-variable, 13-variable, 5-variable, and 4-variable, slightly increased, resulting in 0.95, 0.95, 0.94, and 0.93, respectively. Detailed quantitative results of AUROC, ACC, SEN, SPE, FPR, and PPV for each model are presented in Table 4. Notably, with a cut-off value of 0.5, models 1–4 had an excellent accuracy of over 85%, a sensitivity of over 80%, and a specificity of over 87%. Similarly, models 5–8 maintained excellent accuracy over 85%, sensitivity over 80%, and specificity over 88%. The performance metrics of the 2-variable model and 4-variable model were universally comparable to those of any other models. The ROC curves of models 1–4 and models 5–8 in the training set are shown in Figure 3(a) and (d).

**Table 4:** Performance of models for differentiation of patient groups of FLD by ANN

Parameter	Model 1 (18 variables)			Model 2 (11 variables)			Model 3 (3 variables)			Model 4 (2 variables)			Model 5 (20 variables)			Model 6 (13 variables)			Model 7 (5 variables)			Model 8 (4 variables)		
	Training set	Validation set	Testing set	Training set	Validation set	Testing set	Training set	Validation set	Testing set	Training set	Validation set	Testing set	Training set	Validation set	Testing set	Training set	Validation set	Testing set	Training set	Validation set	Testing set	Training set	Validation set	Testing set
Threshold	0.5	0.5	0.5	0.5	0.5	0.5	0.5	0.5	0.5	0.5	0.5	0.5	0.5	0.5	0.5	0.5	0.5	0.5	0.5	0.5	0.5	0.5	0.5	0.5
AUROC	0.95	0.92	0.91	0.94	0.93	0.91	0.93	0.92	0.91	0.92	0.91	0.89	0.95	0.93	0.92	0.94	0.94	0.92	0.94	0.92	0.92	0.93	0.92	0.91
SEN	84.7%	81.3%	83.3%	86.2%	85.6%	82.9%	82.4%	82.0%	78.2%	80.0%	80.6%	77.0%	85.0%	83.5%	87.4%	82.9%	79.1%	83.5%	82.6%	81.1%	83.0%	80.7%	79.2%	79.8%
SPE	90.1%	89.3%	85.5%	87.6%	86.3%	83.8%	87.3%	88.1%	87.0%	88.1%	85.6%	85.6%	89.5%	89.0%	84.2%	89.7%	90.7%	87.0%	89.0%	87.7%	85.8%	88.7%	88.6%	86.4%
ACC	88.0%	86.2%	84.1%	87.1%	86.1%	83.2%	85.4%	85.6%	81.6%	85.1%	83.6%	80.3%	87.8%	86.9%	86.2%	87.0%	86.2%	84.8%	86.5%	85.2%	84.1%	85.6%	85.0%	82.3%
FPR	9.9%	10.7%	14.5%	12.4%	13.7%	16.3%	12.8%	11.9%	13.0%	11.9%	14.4%	14.4%	10.5%	11.0%	15.8%	10.3%	9.3%	13.0%	11.0%	12.3%	14.2%	11.3%	11.4%	13.6%
PPV	84.5%	82.8%	90.3%	81.6%	80.0%	89.3%	80.4%	81.4%	90.8%	81.1%	78.1%	89.7%	83.8%	82.9%	90.0%	83.7%	84.4%	91.3%	82.7%	80.8%	90.5%	82.0%	81.6%	90.5%

Abbreviations: AUROC, area under the receiver operating characteristic curve; ACC, accuracy; SEN, sensitivity; SPE, specificity; FPR, false positive rate; PPV, positive predictive value. There were 1,717 (training set), 737 (validation set), and 1,041 (testing set) patients with FLD and 2,698 (training set), 1,157 (validation set), and 640 (testing set) patients with non-FLD.

### 4.2 Validation group

When the ANNs were evaluated in the validation group, the models in predicting FLD yielded AUROCs of 0.92, 0.93, 0.92, and 0.91 among models 1–4, while models 5–8 yielded AUROCs of 0.93, 0.94, 0.92, and 0.92. Although the AUROC values were universally slightly lower than those obtained in the training group, they were still regarded as strong indicators of model performance. The quantitative results corresponding to the validation are summarized in Table 4. The ROC curves yielded by the models in the validation set are plotted in Figure 3(b) and (e) for the first four and last four models.

### 4.3 Testing group

The performance provided by the 18-variable model, the 11-variable model, and the 3-variable model in the testing group had an overall high predictive ability with AUROC of 0.91. Although the 2-variable model’s performance was slightly lower than that of the other three models (0.89 vs 0.91), it showed a comparable performance among them (Table 4). When age and gender were added into the models, the resulting 20-variable, 13-variable, 5-variable, and 4-variable models exhibited AUROCs of over 0.91 in the testing group. This slight improvement in AUROC values highlights the added predictive value of including age and gender. In particular, despite the TBIL, DBIL, URE, and CRE, the other serum variables in the testing set were significantly different from those in the training set and the validation set (Table S1), which demonstrated the well generalization ability of the ANNs in turn. Especially, it was indicated that the proposed ANNs exhibited high predictive ability in FLD screening. The ROC curves of these methods in the testing set are presented in Figure 3(c) and (f) for the first four and last four models.

## 5 Discussion

FLD has become a significant global health concern due to its increasing prevalence and widespread occurrence across different age groups. In recent years, the utilization of machine learning models, particularly ANNs, has presented a unique opportunity to enhance the comprehensive management of FLD. These models have demonstrated their potential in improving the risk prediction and diagnosis of FLD [18]. By analyzing medical variables, machine learning models, such as ANNs, offer an efficient approach to uncover hidden relationships among variables that may otherwise go unnoticed. Their ability to extract hidden associations from complex and diverse clinical datasets has been well established [5].

The confusion matrix analysis showed that the ANNs using serum variables could achieve their predictive purpose. In the present study, we compared the combination of the serum-based panels as predictors, respectively. Eventually, the 18-variable model, the 11-variable model, the 3-variable model, and the 2-variable model were developed, all having the same two core predictors, ALT and TG. Whereas, compared to the 2-variable model, the 3-variable model included an extra predictor, HDL. What is more, to assess the increased value of demographic factors, we incorporated age and gender into our existing models. This resulted in a 20-variable model, a 13-variable model, a 5-variable model, and a 4-variable model.

ANNs utilizing serum variables have exhibited promising predictive capabilities in the field of FLD. The evaluation of ROC curves has demonstrated high predictive accuracies for models 1–4 in the training, validation, and testing sets. The AUROCs were 0.91, 0.91, 0.91, and 0.89 among the testing sets of the first four models, respectively, which further confirmed the robustness of ANNs employing serum variables as a reliable method for FLD prediction. Notably, the primary variables influencing FLD prediction were observed to be ALT and TG. The 2-variable model exhibited comparable performance to the 18-variable, 11-variable, and 3-variable models, indicating that an effective distinction between FLD and non-FLD patients can be achieved through a simplified approach.

Interestingly, when age and gender were included in the first four models, it resulted in AUROCs of 0.92, 0.92, 0.92, and 0.91 for the 20-variable, 13-variable, 5-variable, and 4-variable models among the testing sets, respectively. The slight improvement in AUROCs highlights the enhanced predictive performance with the inclusion of age and gender, indicating that these two factors are critical elements in effectively distinguishing between FLD and non-FLD patients. Despite this enhancement, the 4-variable model with age and gender demonstrated comparable performance to more complex models, reinforcing its potential as a superior option for accurately and effectively predicting FLD. Consequently, our findings suggest that the implementation of an uncomplicated model could serve as a superior option for accurately and effectively predicting FLD. These results warrant consideration for the development of an appropriate predictive system in this domain.

Attempts have been made to enhance the performance of FLD prediction by incorporating additional variables through machine learning techniques. As early as 2014, Vanderbeck et al. [19] employed a support vector machine algorithm with handcrafted features to identify and quantify various structures on scanned Hematoxylin and Eosin slides from nonalcoholic FLD (NAFLD ) and healthy liver biopsies, achieving an overall accuracy of 89%. Roy et al. [20] developed a U-Net architecture algorithm that effectively segmented and quantified hepatic steatosis. Lin et al. [21] utilized multivariate analysis incorporating sex, age, TG, BMI, TC, and ALT, indicating that multinomial logistic regression (LR) exhibited the highest predictive power, with an accuracy rate of 72.6% for first-degree FLD and 62.3% for second- and third-degree FLD. Islam et al. [22] developed four classification models – Random Forest (RF), Support Vector Machine (SVM), ANN, and LR for FLD prediction, with LR yielding the best results (76.3% accuracy, 74.1% sensitivity, 64.9% specificity). In addition, Wu et al. [23] created four classification models – RF, Naïve Bayes (NB), ANN, and LR – to evaluate the optimal predictive clinical model for FLD, where the RF exhibited superior performance with 10-fold cross-validation, achieving an accuracy of 86.48% and an AUROC of 0.925. This model incorporated 10 clinical values, including age, gender, systolic blood pressure, diastolic blood pressure, abdominal girth, glucose AC, TG, HDL-C, AST, and ALT. Okanoue et al. [18] reported that the artificial intelligence/neural network system utilizing 11 medical values (including age, gender, height, weight, waist circumference, AST, ALT, GGT, cholesterol, TG, and PLT) had well efficacy in diagnosing NAFLD (AUROC > 0.950). Overall, these studies showed the potential of machine learning technology for identification in patients with FLD.

Although the application and assessment of machine learning had been explored for the recognition of FLD using tongue images [24], liver biopsy images [19], US [25,26], clinical data [17,27,28], and a combination of US and clinical data [17,27,29], a promising model for FLD prediction only on the basis of serum data with very few variables has seldom been applied in routine clinical care. Even though differences in variables were observed between patients with and without FLD, the discrepancies in test outcomes were not significant enough to utilize a single biomarker as an independent predictor of FLD. Therefore, we employed ANN to integrate only serum variables alone and in combination with age and gender that accurately classify patients at high risk of FLD during examination. Particularly, the 2-variable model using TG and ALT and the 4-variable model, which incorporated age and gender into the 2-variable model, were found to be with good performance in the training set, the validation set, and the testing set. This fully demonstrated the superiority of the neural network and the well generalization ability of the ANNs, which were with sufficient accuracy to be usefully employed as a reliable and user-friendly tool for identifying FLD.

However, it is important to acknowledge the limitations of this study. One possible limitation is the choice of modeling methods, which can significantly impact the accuracy of disease prediction models. Therefore, future research aims to develop more advanced neural network models combined with image analysis to facilitate the diagnosis of FLD. Additionally, the data collection was limited to one medical center. There may be selection bias in the data selection process, which needs to be handled carefully to ensure the reliability and validity of the research results. Multicenter datasets should be sought to further improve the reliability and clinical usability of the constructed models. Further research in this area, exploring the utilization of ANN and other machine learning technologies, holds promise for improved results and enhanced preventive healthcare.

## 6 Conclusion

In conclusion, this study successfully developed an ANN-based variables integration model by constructing different FLD prediction models, to integrate the information of only serum variables alone and alongside age and gender for accurately predicting patients with the FLD, allowing patients to be tested for just two serum markers (ALT and TG) to determine if further diagnostic tests are needed, which avoids unnecessary and excessive medical treatment, so that patients can receive appropriate treatment at an early stage. We confirmed the application value of the prediction of FLD, providing strong support for the follow-up application in disease prediction. The FLD prediction model combined with serum variables having well repeatability, reproducibility, and generalization ability, is worthy of further exploration. We anticipate the functionality of the system to provide significant patient benefits.

## Abbreviations

ACC: Accuracy
ANN: Artificial neural network
AUROC: Area under the receiver operating characteristic curve
ALT: Alanine aminotransferase
AST: Aspartate aminotransferase
ALB: Albumin
ALP: Alkaline phosphatase
CRE: Creatinine
DBIL: Direct bilirubin
FLD: Fatty liver disease
FPR: False positive rate
GLB: Globulin
GGT: Gamma-glutamyltransferase
HDL-c: High-density lipoprotein cholesterol
LDH: Lactic dehydrogenase
LDL-c: Low-density lipoprotein cholesterol
LR: Logistic regression
NB: Naïve Bayes
NAFLD: Nonalcoholic fatty liver disease
PPV: Positive predictive value
RF: Random Forest
SVM: Support Vector Machine
SEN: Sensitivity
SPE: Specificity
TC: Total cholesterol
TG: Triglyceride
TP: Total protein
TBIL: Total bilirubin
UA: Uric acid
URE: Ureophil
US: Ultrasound

## Supplementary Material

Supplementary Table
