# Supplementary material for: Laboratory variables‐based artificial neural network models for predicting fatty liver disease: A retrospective study
Source: Open Med (Wars). 2024 Sep 13;19(1):20241031. doi: 10.1515/med-2024-1031 (PMC11406433; doi:10.1515/med-2024-1031)
Supplement: Supplementary Table [file med-2024-1031-sm.pdf]

Supplementary material

Laboratory variables-based artificial neural network models for predicting fatty liver disease: a retrospective study.

Table S1: Comparing input and output variables in the three sets

Variables	Training set (*N* = 4,415)	Validation set (*N* = 1,894)	Testing set (*N* = 1,681)	*p*-value[1]	*p*-value[2]
Male gender	2078(47.0%)	905(47.7%)	863(51.3%)	0.352	0.010
Median age(years)	36.8(±7.8)	36.6(±7.9)	39.4(±10.4)	0.602	<0.001
TC(mmol/L)	4.6(±0.9)	4.6(±0.8)	5.0(±0.9)	0.266	<0.001
TG(mmol/L)	1.6(±1.9)	1.6(±1.4)	1.8(±1.7)	0.382	<0.001
HDL(mmol/L)	1.4(±0.4)	1.4(±0.4)	1.3(±0.4)	0.943	<0.001
LDL(mmol/L)	3.0(±0.8)	3.0(±0.8)	3.1(±0.8)	0.451	<0.001
ALT (U/L)	24.6(±25.3)	23.5(±22.5)	29.2(±25.8)	0.092	<0.001
AST(U/L)	20.8(±13.0)	20.3(±11.1)	20.7(±10.2)	0.178	<0.001
TP(g/L)	75.1(±3.9)	75.2(±3.8)	75.5(±4.0)	0.489	0.001
TBIL(μmol/L)	11.4(±5.4)	11.5(±5.5)	11.5(±5.1)	0.402	0.690
DBIL(μmol/L)	4.3(±1.6)	4.3(±1.6)	4.4(±1.6)	0.848	0.867
A/G	1.9(±0.3)	1.9(±0.3)	1.8(±0.3)	0.311	0.023
GLB(g/L)	26.6(±3.5)	26.7(±3.4)	28.1(±3.7)	0.445	<0.001
ALB(g/L)	48.5(±2.6)	48.5(±2.6)	47.4(±2.5)	0.983	<0.001
ALP(U/L)	68.8(±21.8)	69.8(±24.1)	72.8(±20.5)	0.154	<0.001
GGT(U/L)	29.8(±34.8)	29.2(±30.8)	36.0(±34.6)	0.578	<0.001
LDH(U/L)	160.1(±25.9)	160.7(±26.6)	167.1(±28.7)	0.356	<0.001
URE(mmol/L)	4.7(±1.2)	4.7(±1.2)	4.7(±1.1)	0.505	0.380
CRE(μmol/L)	72.0(±17.9)	72.3(±15.5)	73.0(±15.1)	0.615	0.153
UA(μmol/L)	335.4(±96.2)	335.8(±97.3)	354.5(±99.6)	0.871	<0.001

Results are shown as the mean ± SD. *p*-value[1] were calculated between the data of the training set and the validation set. *p*-value[2] were calculated among the data of the training set, the validation set and the testing set.
